# Adverse effects of heat shock in rice (*Oryza sativa* L.) and approaches to mitigate it for sustainable rice production under the changing climate: A comprehensive review

**DOI:** 10.1016/j.heliyon.2024.e41072

**Published:** 2024-12-07

**Authors:** Mohammad Mobarak Hossain, Sharif Ahmed, Mohammad Saiful Alam, Akbar Hossain

**Affiliations:** a On-Farm Research Division, Bangaldesh Wheat and Maize Research Institute, Nashipur, Dinajpur, 5200, Bangladesh; b International Rice Research Institute Bangladesh Office, Banani, Dhaka, 1213, Bangladesh; c Bangladesh Jute Research Institute, Manik Mia Avenue, Dhaka, 1207, Bangladesh; d Soil Science Division, Bangaldesh Wheat and Maize Research Institute, Nashipur, Dinajpur, 5200, Bangladesh

**Keywords:** Global warming, High temperature, Heat tolerance, Growth regulators, Cultural practices, Genetic engineering

## Abstract

Heat shock, a transient exposure to high temperatures, is a substantial hazard to rice (*Oryza sativa* L.) production and sustainability. The objective of this review paper is to summarize the impact of heat shock on rice and explore approaches to mitigate its adverse effects to achieve sustainable production. Rice is a staple food for billions of people globally and is extremely sensitive to heat shock. Higher temperatures disturb various physiological and biochemical processes, resulting in decreased growth, development, and ultimately lower grain yield. Heat shock negatively affects important agronomic traits, such as panicle differentiation, pollen viability, fertilization, grain filling, and, ultimately, grain quality. To manage heat shock and sustain rice production, several strategies have been explored, such as modifications to sowing schedules, the substitution of heat-tolerant cultivars for sensitive genotypes, and the use of growth regulators. To improve rice under heat shock, various approaches could be taken: (1) cultivating cultivars that flower early in the morning by adjusting sowing/planting times, modified irrigation, and fertilization; (2) inducing acclimation via growth regulators and organic stimulants and chemicals; (3) breeding genetically resistant cultivars through the integration of appropriate genes; and (4) genetic modification techniques for heat-shock tolerance. Overall, effectively managing heat-shock stress in rice requires a comprehensive strategy that includes developing and using heat shock-tolerant cultivars, adopting suitable cultural practices, utilizing external substances, and applying biotechnological tools. Implementing these strategies collectively will help achieve sustainable rice production in the face of increasing heat-shock conditions.

## 1 Introduction

The geographical distribution of crop species is significantly influenced by the ambient temperature [1]. The seasonal growth, development, and reproduction of plants are all influenced by the ambient temperature, and each species has distinct optimal temperatures for these processes [2]. However, global warming has become an issue that hinders the optimal temperature because of population expansion and industrial development in recent eras [3]. The ADB and IPCC predict that by 2100, the global mean surface temperature will rise by 3–5 °C. Southeast Asia is anticipated to experience an average yearly increase in temperature of 0.7–0.9 °C [4,5]. According to global climate models, there is a >90 % chance that extreme seasonal tropical temperatures recorded over the last 100 years will exceed most temperatures by the end of the twenty-first century [6]. Heat waves are becoming more frequent and longer-lasting, impacting global crop production [7]. For every 1 °C increase in the global mean temperature, the yields of rice, maize, wheat, and soybean are projected to decrease by 3.2, 7.4, 6.0 and 3.1 %, respectively [8]. To meet the needs of nine billion people by 2050, food production must increase by an additional 70 % [9].

Rice is the primary food for more than half of the global population, contributing to 80 % of caloric requirements globally [10]. Temperatures between 25 and 35 °C are ideal for rice cultivation, and rice thrives in regions with moderate temperatures [11]. A minimum of five reproductive days with temperatures exceeding physiological levels will impact sixteen percent of rice-harvested land by 2030; by 2050, this proportion is projected to increase to twenty-seven percent [12]. However, to meet the demand of the growing population, ∼30 % more rice is needed globally by 2050 [13].

Heat shock (HS) is characterized by a prolonged and consistent increase in temperature above a certain threshold, which results in irreversible damage to the growth and development of plants [3,14]. It has detrimental effects on the reproductive and developmental phases of rice. These include impeding pollination, causing dehiscence and spikelet sterility, and reducing plant height and root elongation [15]. HS is anticipated to cause a 41 % decline in rice yield by the end of this 21st century [16]. In addition, HS not only negatively affects rice growth but also impairs grain quality, resulting in substantial economic losses. In a study in South China, Shi et al. [17] reported that postheading HS from 1981 to 2010 reduced the rice yield by 1.5–9.7 %. According to Lyman et al. [18], every 1 °C increase in average temperature reduces total milled rice by 8 %, head rice by 14 %, and milled rice by 11 %. This lowered the paddy yield by 6 %. Bangladesh experiences heat stress when temperatures exceed 36 °C, compared to the normal 33 °C in April. High temperatures, minimal precipitation, and low humidity have hindered spring rice growth. In early April, when temperatures reached 36 °C for two consecutive days, over 36 districts were affected [11]. Climate scientists warn that food supplies in Bangladesh are at risk, leading to rice producers in the country facing losses of US$39 million.

Thermotolerant rice varieties can overcome the deleterious consequences of HS [[19], [20], [21]]. Recent research on HSs has mainly concentrated on reproductive processes and grain production. Evaluations have shown a lack of information regarding the consequences of HS. This review outlines the impacts of HS on different developmental phases of rice while also exploring potential strategies and approaches to improve rice tolerance to HS.

## 2 Methodology

A thorough literature search was conducted from January 2022–December 2023, utilizing digitized repositories such as Google Scholar, Scopus, PubMed, and Web of Science. Literature published from 1999–2023 was used to discuss the issue. Variations of the following search phrases were employed: “heat shock”, “rice cultivation”, “*Oryza sativa*”, and “sustainable production”. We included primary research articles, meta-analyses, and review papers that examined the effects of heat shock (HS) on rice cultivation and sustainable production management practices. A total of 127 studies were analysed, and the results were synthesized in this article. NonEnglish articles or those without a clear emphasis on the effects of HS on rice were omitted from the review. The data obtained from the selected papers were systematically organized by key topics. These topics include the physiological responses of rice plants to heat stress, the effects of heat stress on rice quality and yield, and various strategies for managing heat stress. A comprehensive review of the literature was conducted to present the current level of understanding in this specific field. The authors addressed practical concerns and suggested future research directions, leveraging their combined expertise in rice farming and heat stress physiology, along with secondary data sourced from published literature. Valuable insights were gained through collaboration with specialists and discussions with experts in the field. In addition to the author's insights and experiences, the data from the literature review were integrated to provide a comprehensive assessment of the effects of HS on rice farming and suitable management techniques for sustainable production. Priority was given to the identification of knowledge gaps and the proposal of prospective future research domains.

## 3 Temperature requirements for rice growth and development

Rice has a distinct temperature preference because of its thermophilic nature [19]. Indica rice needs 25–35 °C for cultivation, whereas japonica rice needs 20–33 °C. The critical high temperature commonly exceeds 35 °C. However, there is variation in rice development from one phase to the next (Table 1) [20,21].

**Table 1:** Temperature (°C) requirements for rice growth and development.

Different growth stages	Ambient Temperature	Threshold Temperature	
		Minimum	Maximum
Germination of seeds	20–35	10	45
Emergence and establishment of seedlings	25–30	12–13	35
Formation of roots	25–28	16	35
Elongation of leaves	31	7–12	45
Development of tillers	25–31	9–16	33
Heading/Anthesis	30–33	22	35
Maturity	20–25	12–18	30

The development phase of rice depends on factors such as the variety, duration of the critical temperature, growth conditions, and daily variations [22]. The ideal conditions for germination and seedling development are 25–35 °C [23]. Thermal conditions of 12–13 °C and 28–29 °C at night and during the day, respectively, are critical for reproduction [24]. An increase in temperature beyond the optimal range is detrimental to the development and growth of rice.

## 4 How does heat shock affect the growth and development of rice?

Elevated temperatures adversely affect most rice growth stages, including emergence, ripening and harvesting. The severity of potential crop damage depends on the developmental stage during which the plant encounters heat stress and the duration of heat stress. Krishnan et al. [21] reported that rice plants exhibit HS at temperatures greater than 35 °C, depending on the cultivar and growth stage. HS harms seedlings during emergence. HS slows germination and seedling growth. The plant height, tiller number, and dry weight may decrease during vegetative growth. According to previous reports, the rice dry weight at 35/25 °C was only 1/6 that at 30/25 °C. The leaves discoloured and dried for 2 days at 45/25 °C, after which they died 9 days later. Even for photoinsensitive cultivars, temperatures greater than 26 °C shortened the period of heading. Increasing the temperature from 29/21 to 37/29 °C reduced the tiller number by 10 %. The vegetative and reproductive phases are twice as short at 29/21 °C. HS before or during anthesis decreases seed set [25] and inhibits spikelet fertilization, often resulting in sterile grains, ranging from a few empty spikelets to near-total yield loss. This may have resulted from the loss of pollen activity, pollen germination, and floret fertility. With increasing temperature and duration, the rate of seed setting decreases. In addition, the dehiscence rate, pollen fertility rate, number of spikelets per panicle, seed-setting rate, 1000-grain weight, and grain yield are significantly diminished by HS during meiosis [26]. The grain yield significantly decreased after 20 days of heading when the temperature exceeded 28 °C [27]. Day/night temperatures exceeding 28/21 °C result in a 10 % reduction in grain production for each 1 °C increase [21].

**Fig. 1:**
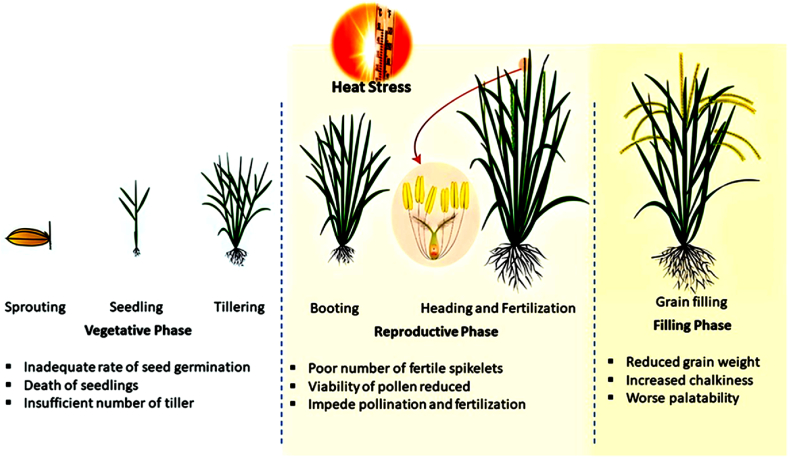
Effects of heat shock on different phases of rice growth and development.

The authors also reported that daytime temperatures greater than 40–41 °C during anthesis can lead to 40–60 % sterility and potentially result in no grain production. High temperature (ambient + 4 °C) leads to a reduction in seed set and panicle weight. The reduction in panicle weight relative to the green leaf area indicates a source/sink ratio problem. HS may reduce sink capacity due to an increase in sterile spikelets and decreased starch synthesis, leading to a lower 1000-grain weight [28]. Elevating the nighttime temperature from 18 to 30 °C diminishes head rice yields, grain dimensions, and amylase content [29]. It diminishes grain width and disrupts grain filling, resulting in chalkiness in grains and starch, resulting in a relatively high gelatinization temperature [30]. HS produces a greater number of fractured grains at relative humidity levels ranging from 25 % to 85 %. This also results in cooked rice grains being harder, less sticky, exhibiting reduced leaching of starch components, particularly amylase, and having a rougher texture, increasing the maximal viscosity, disintegration, and hardness-to-adhesion ratio of cooked rice [31]. The effects of HS on different growth phases are illustrated in Table 2, Fig. 1 and the subsequent sections.

**Table 2:** The impact of heat shock on various phases of rice growth.

Growth Phases	Thermal Threshold (°C)	Symptoms	References
Emergence	40	Inhibition and reduction in emergence	[32]
Seedling	35	Poor development of the seedling	[21]
Tillering	32	Height and tillering reduction	[21]
Booting	41	Reduced quantity of pollen grains	[33]
Anthesis	33.7	Dehydration and sterility of the anthers	[15]
Flowering	35	Sterility of flowers	[20]
Grain formation	34	Sterile spikelets	[34]
Grain ripening	29	Reduced grain filling	[21]

### 4.1 Impact of heat shock on the vegetative stage

Seed vigour, a crucial aspect of rice plant establishment, is determined primarily by germination capacity and early seedling growth. Prolonged exposure to elevated temperatures beyond the ideal range of 25–28 °C during the seedling stage can significantly diminish the chance for seed germination and impair the vigour of the seedlings [35]. Rice plants can tolerate temperatures as high as 35 °C during the day and 25 °C at night during the vegetative stage. However, temperatures exceeding this critical threshold can lead to reductions in plant height, tiller number, and overall dry matter deposition.

During the tillering stage, when rice plants actively develop new shoots, exposure to HS causes various visible changes in their morphology. These changes include wilting, leaf curling, and yellowing and a reduction in the number of shoots and overall plant biomass [36].

High temperatures can affect the photosynthetic machinery of rice plants, reducing their chlorophyll content and photosynthetic efficiency, ultimately impacting biomass accumulation. In a previous study, rice was subjected to temperatures 3.6–7.0° higher than the ambient conditions in a controlled-temperature chamber. The results revealed a substantial decline in photosynthesis, ranging from 11.2 % to 35.6 %, during the period between the heading and middle ripening stages [27]. The decline in photosynthesis is attributed to alterations in the structure of thylakoid membranes, particularly the disruption of granum stacking within chloroplasts [37]. Experiments conducted in controlled environments using free air temperature increment (FATI) chambers and temperature gradient tunnels (TGTs) demonstrated significant decreases in several key rice plant growth parameters under heat stress conditions. These parameters included reduced growth duration, decreased leaf area index, lower biomass accumulation, diminished yield, and smaller harvest indices. Conversely, spikelet sterility and stability indices significantly increased [38]. Compared with *Indica* rice, HS had a greater effect on the quantity of tillers and panicles in *Japonica* rice. Under HS conditions, tiller number serves as a valuable marker for selecting thermotolerant rice cultivars.

### 4.2 Effect of heat shock on the reproductive phase

HS poses a more significant threat to rice plants during their reproductive phase, potentially leading to greater yield losses than during the vegetative stage [21]. HS during this period can lead to various detrimental effects, including inhibited panicle and spikelet development, deformities in floral organs, and reduced spikelet size and quantity. Spikelet reduction is attributed to the degeneration of spikelets at the panicle tips [39]. HS during the meiosis phase of the pollen mother cell at the anther development stage induces untimely deterioration of tapetal cells. This disruption impedes the ability of microspores to acquire nutrients and hinders pollen wall formation, ultimately resulting in pollen grain abortion [40]. During the reproductive phase of rice, the anthesis stage is the most susceptible to HS, followed by microgametogenesis. Temperatures above 35 °C for approximately five days during anthesis can induce high spikelet sterility in rice and failure to produce seeds [41]. HS also leads to substantial reductions in pollen tube diameter and length, resulting in impaired pollen grain germination. Consequently, efforts have focused on enhancing the tolerance of the male reproductive system to warmer climates, as it is the primary contributor to spikelet sterility under HS. HS exposure during anthesis can have irreversible effects, causing a decline in panicle dry weight even if environmental conditions improve subsequently. Subjecting rice plants to high temperatures (41 °C during the day and 30 °C at night) for ten days during the pollen mother cell meiosis stage resulted in a dramatic 78.8 % reduction in pollen viability and a substantial 48.5 % decrease in the seed setting rate compared with those under control conditions (30 °C during the day and 24 °C at night) [42,43]. Arshad et al. [44] reported that HS disrupts anther dehiscence, leading to decreased pollen dispersal and fewer pollen grains on the stigma, as well as reduced starch biosynthesis in developing grains, ultimately resulting in lower starch accumulation and grain yield.

The spikelets became sterile when the temperature rose above 32 °C for one night just before anthesis, even when there was no high temperature during the day. Three-night exposure to high temperatures of ≥30 °C greatly reduced the number of fertile grains. This value is much lower than the stated threshold of 35 °C for heat-induced sterility during the day. In addition, the sterility caused by high daytime temperatures is exacerbated by high nighttime temperatures [45].

In their study, Abeysiriwardena et al. [46] reported that rice grains almost completely became sterile when they were exposed to high temperatures (35 °C day/30 °C night) and high relative humidity (RH) (85–90 %) at heading. However, lowering the RH by 30 % made the grains less sterile but did not significantly influence the percentage of fully filled grains, which was very low at both very high and low RH levels. For a normal temperature of 30 °C during the day and 25 °C at night, the lowest recorded 13.8 % grain sterility at normal RH (65–70 %), which was approximately two times greater at very low RH (35–40 %).

A model for predicting spikelet sterility by high temperature was used by Nguyen et al. [47] to demonstrate that spikelet fertility decreases logistically as the temperature increases during meiosis and flowering. The fertility responses to high temperatures on the day of spikelet meiosis and at the time of spikelet anthesis were effectively correlated with the logistic functions of the degree of heating above 31 °C 12 days prior to spikelet anthesis and the atmospheric temperature at the time of spikelet flowering, respectively. During meiosis, the number of heating hours resulting in 50 % spikelet sterility was greater in the *japonica* cultivar "Hwaseongbyeo" than in the *Tongil*-type cultivar "Dasanbyeo". Conversely, the air temperature that caused 50 % spikelet sterility at spikelet flowering time was greater in *Tongil*-type cultivars than in *japonica* cultivars.

### 4.3 Impact of heat stress on the ripening phase

The ripening stage, as described by Sreenivasulu et al. [48], represents the final phase of a crop plant's growth and marks the initiation of lipid, protein, and carbohydrate transport and synthesis within seeds. HS exposure during the critical period of grain filling has detrimental effects on the cellular and developmental processes associated with ripening, leading to grain yield and quality reductions. Exposing rice plants to higher temperatures during grain filling often results in various negative effects, including decreased grain mass, hindered grain development, a higher occurrence of white chalky rice, and a milky white appearance [21]. Moreover, high temperatures dramatically reduce the amount of amylose in grains and their overall size. HS experienced after anthesis substantially diminishes test weight, dry matter deposition, and percent grain filling, which ultimately leads to a lower yield [49]. Subedi et al. [50] reported that prolonged elevated temperatures can weaken plants, increasing their susceptibility to pests and diseases and leading to further crop loss.

### 4.4 Impact of heat stress on the photosynthetic efficiency, nutrient uptake, and water relationships of rice plants

High temperatures can cause stress in plants, which can lead to the breakdown of chlorophyll. This reduces the ability of plants to capture light energy for photosynthesis. The enzyme Rubisco, which is important for carbon fixation, becomes less efficient at higher temperatures, reducing the overall rate of photosynthesis. HS often causes stomatal closure to minimize water loss, which in turn limits the uptake of carbon dioxide (CO_2_) and further reduces photosynthetic rates. Additionally, high-temperature stress can destabilize thylakoid membranes in chloroplasts, affecting the electron transport chain and ATP production, both of which are essential for photosynthesis [[51], [52], [53]].

Heat stress can harm the cells of roots, which reduces their growth and function [54]. As a result, plants are less able to take up nutrients from the soil. High temperatures can change the chemistry of the soil, affecting the ability of essential nutrients such as nitrogen, phosphorus, and potassium to dissolve and be absorbed by rice plants [55]. Heat shock can also have a negative effect on the expression and function of transport proteins responsible for nutrient uptake, making it even more difficult for plants to absorb nutrients [56].

Alternatively, high temperatures can lead to excessive transpiration, resulting in water stress, especially when the water supply is inadequate [57]. This can cause wilting and reduce turgor pressure in cells. Even with sufficient soil moisture, heat stress can lead to physiological drought, where the plant cannot access water because of compromised root systems or stomatal closure [58]. Extended heat exposure can result in cavitation (the formation of air bubbles) in xylem vessels, disrupting water transport and leading to inefficient water distribution within the plant [59].

## 5 Approaches to increase rice tolerance to HS

**Fig. 2:**
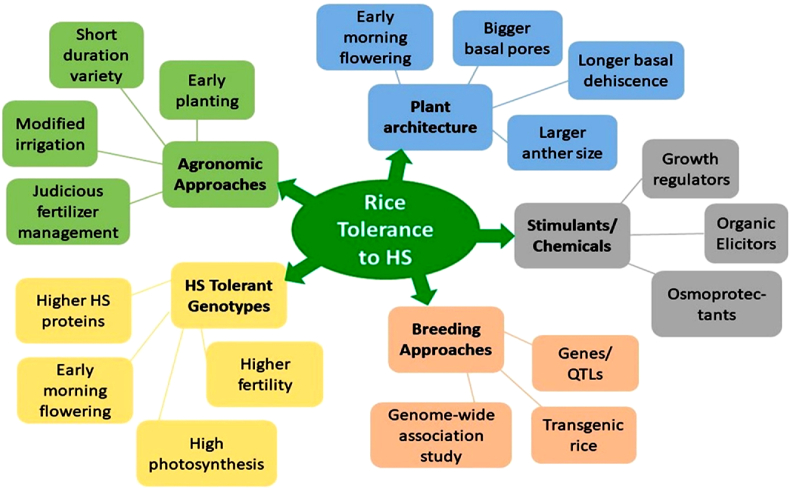
Strategies to increase rice tolerance to HS.

The ability of rice plants to escape, avoid, or endure heat stress is due to various coping mechanisms (Fig. 2). Various strategies that might be used for increasing the HS tolerance of rice are outlined below.

### 5.1 Agronomic management

Principally, agronomic management strategies focus on early seeding/planting of late- or early-maturing cultivars, crop zoning, and modified irrigation system adaptations to avoid HS during grain filling [27,60]. Timely planting of rice varieties is essential to prevent HS during critical growth phases.

Agronomic management encompasses a collection of techniques and practices employed to create favourable conditions for crop growth while minimizing the detrimental effects of HS. Numerous agronomic management strategies emphasize early rice planting, adapting crop selection to specific locations, and modifying irrigation systems [61]. Additionally, these strategies include selecting rice varieties that mature earlier or later to prevent exposure to heat stress during grain development, thus reducing the negative effects of heat stress. The timing of rice planting has a significant impact on how rice plants react to different temperature conditions. In a study by Setiyono et al. [62], substantial yield losses due to HS-induced sterility of spikelets were reported to be alleviated by early planting of rice.

Some studies indicate that adjusting the timing of sowing may alleviate the negative effects of HS on the degradation of rice grain quality [60]. Sowing time adjustment is an effective management strategy for mitigating HS, according to the findings of Zhu et al. [63], who examined the impact of high temperatures on the grain quality of rice genotypes at various sowing times. The authors recommended that farmers create a detailed cropping calendar for the entire year, as changing the planting schedule can negatively impact the previous harvest.

The primary sources of global warming from agriculture are anaerobic flooded systems' CH_4_ gas emissions and aerobic conditions’ N_2_O production. By reducing CH_4_ and N_2_O emissions, adjusting irrigation systems, such as alternate wetting and drying (AWD) for aerobic rice, is an additional technique for mitigating HS [42,64]. To mitigate HS, two potential strategies are to amend the microclimate with shade or incorporate agricultural wastes and manure into the soil to preserve moisture [21]. These data suggest that conservation agriculture farming might be advantageous in this situation. In their study, Shahid et al. [65] reported that the introduction of boron into the soil effectively mitigated the detrimental impacts of heat stress. Furthermore, rice plants subjected to boron treatment presented increased cell membrane integrity, spikelet fertility, and yield. Rice plants can be protected from the detrimental effects of HS (33 °C day temperature at the tillering stage) through the use of biochar at a rate of 40 g kg^−1^ soil. This is achieved by enhancing the chemical, biological, and physiological characteristics of the soil; as a result, the morphological, architectural, and physiological attributes of the roots are improved, as are the shoot uptake and utilization of nitrogen [66].

### 5.2 Heat-tolerant rice cultivars with special phenotypic characteristics

#### 5.2.1 Early morning flowering

One potential strategy for mitigating spikelet sterility is to advance the blooming period during the early morning hours when ambient temperatures are relatively low [21]. The identification of genes that control the time of flowering is highly important for understanding the mechanism underlying heat tolerance and transmitting this trait. In flooded rice environments, early blooming might be used in breeding to confer HS tolerance to rice genotypes [67,68]. In a study, Ishimaru et al. [69]. [48] reported that the early blooming characteristics of the wild rice *Oryza officinalis* were adapted for the *Oryza sativa* cultivar *Koshihikari*. When the temperature was relatively low, the panicle opening of the developed lines, which presented early-morning blooming characteristics, occurred several hours before *Koshihikari*. In the introgression line, spikelet sterility was reduced when the spikelets were exposed to a temperature that increased gradually with time and upon early flower opening. When the panicles were exposed to a temperature of 38 °C during anthesis, both lines presented 60 % spikelet sterility. This finding suggests that the resistance of the genotypes to high temperatures is comparable. Hirabayashi et al. [70] reported that the sterility of spikelets induced by HS could be significantly reduced by incorporating thermotolerant quantitative trait loci (QTLs) into the Indica background. This integration led to a peak anthesis time shift of approximately 2.0 h in comparison with that of the recurrent parents. Nevertheless, these investigations were carried out in a subtropical environment; the agronomic impact and durability of these characteristics in a tropical climate have yet to be explored.

#### 5.2.2 Crop canopy and orientation

The architectural features of plants have a profound impact on their ability to withstand HS. HS tolerance may be imparted by creating and selecting genotypes with an appropriate architecture. Genotypes should feature more leaves around their panicles to shield and shade the anthers, enhance transpiration-induced cooling, and reduce anther transpiration, thereby increasing heat tolerance [3]. Unlike traditional varieties, novel rice varieties whose panicles are enclosed by a canopy of leaves gain significant advantages from the combined cooling effect of transpiration during the critical anthesis stage [16]. A reduction in transpiration from anthers is necessary for pollen grain swelling which is crucial for anther dehiscence. Poli et al. [71] reported that elevated temperatures increased the HS tolerance of N22 and NH219 rice by increasing their height. When the transpiration rate increases simultaneously, an increase in plant height will assist in the avoidance of HS. Additionally, cultivars with asynchronous tiller and panicle growth showed reduced production but relatively high tolerance to heat stress during a critical developmental phase. Asynchronous panicles protect all panicles from high temperatures during their crucial period, allowing them to avoid HS [21]. Nevertheless, these methods were not chosen by breeders because of the extended ripening time and subsequent yield reduction attributed to other causes. Therefore, the development and adoption of semidwarf plant types with superior canopy structures will be crucial for adapting to rising temperatures. Rice flowers during cooler mornings synchronize anthesis with cooler morning hours, modify flowering patterns, or enhance canopy transpiration cooling [72]. Such rice may efficiently avoid HS when exposed to high temperatures during crucial development stages by using efficient transpiration cooling to maintain beneficial microclimate temperatures below critical levels.

#### 5.2.3 Reproductive organs

During the booting and blooming phases, rice genotypes that possess larger anthers and larger basal pores have a relatively high tolerance to HS [73,74]. Floret sterility under high temperatures leads to fewer pollen grains germinating on the stigma due to inadequate anther dehiscence. Rice cultivars with larger anthers produce more germinated pollen grains, compensating for the reduction in pollen grain production caused by HS [53]. HS inhibits the dehiscence of a membrane by impeding the swelling of pollen grains and the subsequent release of pollen [75,76].

### 5.3 Use of different chemicals

#### 5.3.1 Growth regulators

Mohammed and Tarpley [77] found that the negative effects of heat stress from a 32 °C night time temperature on rice were reduced by exogenous salicylic acid (SA), which enhanced dry matter partitioning by up to 16 % compared to the control [78]**.** In another study, Zhang et al. [79] reported that foliar application of SA at 0–50 mmol/L mitigated the detrimental impacts of HS on rice spikelets. This was achieved through increases in the concentrations of some proline, sugars, antioxidative enzymes (APX, CAT and POD), and phytohormones (ABA, GA3 and IAA). These improvements ultimately resulted in increased yield through the increased number of fertile spikelets. In their study, Chang et al. [80] reported that SA treatment increased the class II heat shock protein (HSP) *Oshsp18.0* in rice. This discovery implies that SA could have a substantial influence on the stimulation of HS in rice.

Methyl jasmonate (MeJA) was also reported to promote flowering in the early morning and was found to alleviate HS [60]**.** The earliest flower opening time i**s** increased by 2 h when MeJA is applied [81]. Exactly 1.2 h after MeJA was applied, flower opening commenced [82]. Ascorbic acid (AsA) has also been reported to inhibit the deterioration of chlorophyll and Rubisco and the accumulation of reactive oxygen species (ROS), which together increase the tolerance of rice to HS [83]. The application of brassinosteroids (BRs) reportedly promotes HS tolerance by increasing the production of HSPs, increasing the expression of genes encoding protective enzymes, and stimulating stomatal conductance, net photosynthesis, and the transpiration rate [84,85]**.**

According to study by Tang et al. [86]**,** auxin and IAA are involved in maintaining the spikelet fertility of HS-induced rice. By impeding the malformation of pollen tubes, Zhang et al**.** [83] reported that rice spikelet sterility was reduced with the application of naphthaleneacetic acid. The combined application of (i) AsA, α-tocopherol, MeJA, and BRs; (ii) vitamin C, vitamin E, MeJA, and BRs; and (iii) SA, α-tocopherol, and glycine betaine (GB) has been reported to increase HS tolerance, characterized by increased yield, increased water usage efficiency, and rapid rates of photosynthesis under high day/night temperatures of 35 °C ± 2/28 ± 2 °C [13,77].

#### 5.3.2 Organic elicitors

The heat stress-induced reductions in the rice leaf chlorophyll content, PSII efficiency, photosynthetic water consumption efficiency, and spikelet fertility were mitigated by the application of the organic elicitor CaCl_2_ (10 mM) [78]. As signalling molecules, nitric oxide and hydrogen peroxide control several plant developmental processes, including fertilization, blooming, and resistance to high-temperature stress [59,87].

#### 5.3.3 Osmoprotectants

When applied exogenously, glycine betaine and proline function as osmoprotectants that increase heat tolerance in rice seedlings by safeguarding membranes and rubisco and citrate synthases. Furthermore, they promote pollen germination and spikelet fertility, even when subjected to temperature stresses ranging from 35 to 45 °C [88,89]. According to Tang et al. [90], the application of spermidine after flowering mitigated the detrimental impact of elevated temperatures on japonica rice yield. This was achieved by regulating the activity of antioxidative enzymes involved in photosynthesis and transpiration, which increased the grain filling rate and yield, as well as starch accumulation, as demonstrated by Fu et al. [91]**.**

### 5.4 Breeding approach for heat tolerance

#### 5.4.1 Heat-tolerant rice genotypes

The variation in the responsiveness of rice cultivars to high temperatures suggests the potential for exploring genotypes with increased resistance to elevated temperatures. In a future warmer climate, genetic diversity might be leveraged to screen tolerant genotypes and to generate tolerant and well-adapted cultivars. Rice varieties are now undergoing screening in many Asian nations. For example, 13 *Indica* rice varieties were investigated by Zhang et al. [92] in the middle and lower regions of China at 37/27 °C day/night temperatures and five varieties presented robust HS tolerance, with stable higher yields.

A research investigation conductedby Ezin et al. [93] identified BRIZ-8B and BRIZ-10B among the six varieties with the highest tolerance to HS at 39 °C. These varieties may therefore be suggested to farmers for cultivation in high-temperature environments and utilized in breeding initiatives aimed at enhancing the heat tolerance of rice. Based on the findings of a study by Permana et al. [94], among the 39 germplasms that were evaluated, 'Aikoku' and 'Nagoya Shiro' are viable breeding materials because of their capacity to withstand heat stress when subjected to severe hot water seed treatment at 70 °C. A 10,000-mutant population was subjected to high-temperature treatment (40–45 °C) for 6 h, from the booting stage to the harvesting stage. The results indicated that the mutant line M9962 produced the most fertile spikelets at 78 %, followed by the lines M3181 and M7988, which produced approximately 70 % of the spikelets [95].

In another study, Masuduzzaman et al. [96] studied 1217 rice germplasms from high-temperature regions and found that only 2 % exhibited noticeable heat stress tolerance. Two lines, IR 86991–146–2–1–1 and IR 87606–109–2–2, were identified as blooming HS tolerant and capable of 86 % spikelet fertility even under 35 °C day temperature conditions. HS-tolerant germplasm can be utilized to improve heat tolerance in future rice genotypes by investigating the processes that contribute to this trait. Nevertheless, genotypes that exhibit tolerance towards a certain form of high-temperature stress may not always demonstrate tolerance towards other types of HS [97].

#### 5.4.2 Genetic improvement for heat-tolerant rice

When faced with HS, plants resist the production of special proteins called *heat shock proteins (HSPs)*. These protein superheroes, especially in vital organs, act as bodyguards for the cell's internal functions. They shield essential processes such as photosynthesis and nutrient uptake from the damaging effects of HS. Studies have revealed a close link between the amount of HSPs and a plant's ability to weather heat. Interestingly, some researchers believe heat stress triggers plants to produce "alert molecules" called reactive oxygen species (ROS). These ROS, once thought solely of as troublemakers, might act as signals to increase HSP production, helping plants adapt to HS conditions.

Several genes participate in the production of *HSPs*, which are activated in response to elevated temperatures and play crucial roles in recovery from HS. Modifying HSPs in genetically modified plants offers the potential for improving heat stress resilience and tapping into rice's intrinsic genetic potential. In a study by Katiyar-Agarwal et al. [98]**,** the *HSP101* gene from *Arabidopsis thalinana* [(L.) Heynh.] was introduced into the *Indica* rice variety *Pusa Basmati 1*, resulting in the generation of transgenic rice. Compared with nontransformed rice, this transgenic rice exhibited typical growth and development but displayed superior recovery from HS.

Similarly, recent findings highlight that the overexpression of the rice *OsHSF7* gene in *Arabidopsis thaliana* leads to increased HS tolerance. The survival rate of plants increased from 22 % to 52 % when exposed to 42 °C for 16 h [99]. Another study by Qi et al. [100] revealed that transgenic rice with the *mtHsp70* gene presented increased HS tolerance. This was evidenced by reduced cell death, restored mitochondrial membrane potential, and decreased ROS production. Nonetheless, only a limited number of studies have shown the tolerance of transgenic rice to HS [60], which is presented in Table 3.

**Table 3:** QTLs and transgenic rice that regulate heat tolerance.

QTLs	Transgenic rice	Features	References
*AtHsp101*	Pusa basmati	Enhance HS tolerance	[98]
*DPB3-1*	*Oryza sativa* L.	HS inducible genes were upregulated	[101]
*fad7*	*Oryza sativa* L.	Silencing of fatty acid desaturase genes	[102]
*mtHsp70*	Nipponbare	Suppress death of the programmed cell	[100]
*OsGSK1*	Dongjin	Enhance HS tolerance	[103]
*OsWRKY11*	Sasanishiki	Increase desiccation tolerance and survival rate of green parts	[104]
*rbcS*	*Oryza sativa* L.	Increase rubisco and photosynthesis in rbcS-sense lines	[105]
*RCA*	*Oryza sativa* (Indica)	Improves growth and yield	[106]
*SBPase*	Zhonghua11	Increase SBPase tolerance	[107]
*sHSP17.7*	Hoshinoyume	Enhance heat, UV-B, and drought tolerance	[108]
*Spl7*	Spl7 mutant	Encodes a HS transcription factor protein	[109]

#### 5.4.3 Gene/QTL introgressions for varietal development

Heat tolerance in rice is not governed by a single gene but rather by a network of genes. QTL (quantitative trait locus) mapping is a technique that has shown promise in elucidating the genetic underpinnings of HS tolerance. Numerous QTL mapping studies have explored heat tolerance, especially spikelet fertility, during key stages of rice development such as booting, flowering, and grain filling. As a result, QTLs associated with thermotolerance have been identified and validated, particularly across developmental stages such as seedling, booting, flowering, and grain filling. Among the QTLs discovered, those located on chromosomes 1 and 4 hold the most promise for increasing HS tolerance [15].

Rice HS tolerance is a quantitative characteristic governed by several genes. While establishing a clear correlation between plant phenotypes and the genes responsible for HS tolerance is challenging, marker-assisted selection serves as a valuable method to generate cultivars that are resistant to HS [110,111]. Rice heat tolerance has been linked to specific QTLs via molecular markers and linkage maps. Research on QTLs linked to HS tolerance in rice, spanning from germination to reproduction, may facilitate the development of HS-tolerant rice varieties by expediting rice breeding for HS tolerance [112]. A study by Wahid et al. [37] emphasizes the importance of cultivating varieties that can endure environmental shocks while sustaining economic productivity to effectively enhance agricultural output. In pursuit of this objective, it is critical to ascertain the genes or QTLs that govern HS tolerance, subsequently integrating them with attributes such as high yield and satisfactory grain quality [72].

Various HS-tolerant rice varieties, including *966, N22, Milyang 23*, and *Giza 178*, possess the *qHTSF4.1* located on chromosome 4. Moreover, a major QTL, OsHTAS, on chromosome 9 has been cloned and shown to confer tolerance to temperatures up to 48 °C in rice seedlings [113]. Additionally, researchers have identified numerous QTLs associated with seedling growth under high-temperature conditions. One such QTL, *RLHT5.1*, which is specifically linked to root length under such conditions, significantly contributes to the overall phenotype [36].

To increase the resistance of rice to HS, the Improved *White Ponni* rice variety was modified with the QTLs *qHTSF1.1* and *qHTSF4.1* derived from *Nagina 22* via marker-assisted breeding procedures, and this variety presented increased percentages of fertile grains when subjected to HS at the flowering stage. These QTLs have been confirmed to provide a vital function in preserving membrane integrity and yield under HS conditions [95,114]. Moreover, Table 4 contains a list of genes compiled by Shen et al. [115] that may encourage rice plants to resist HS.

**Table 4:** Genes linked to rice HS tolerance.

Genes	Function	Reference
*EMF1*	Early ﬂowering	[116]
*HES1*	Maintain chloroplast function	[117]
*HTS1*	Maintain membrane stability and chloroplast integrity	[118]
*HTH5*	Affects seed setting rate	[119]
*OsBHT*	Participate in heat shock response as a molecular chaperone	[120]
*OsCAO1*	Control leaf senescence	[121]
*OgTT1*	Remove denatured cytotoxic proteins	[122]
*OgTT2*	Protect the biosynthesis of wax	[123]
*OgTT3.1*	Vacuolar degradation of ubiquitinate *TT3.2*	[124]
*OgTT3.2*	Protect chloroplast from damage	[124]
*PSL50*	Control premature leaf senescence	[125]
*SLG1*	Control the concentration of thiolated tRNA	[126]
*TOGR1*	Regulate rRNA homeostasis	[127]

## 6 Conclusions and future directions

Heat shock poses a significant risk to rice farming in regions with high temperatures. This review paper highlights the adverse effects of heat shock on the growth, development, yield, and quality of rice. Climate change-induced heat stress is becoming an increasingly serious concern for rice farmers worldwide. However, various strategies can be employed to manage heat shock stress and ensure sustainable rice production. The implementation of management strategies, the selection of tolerant rice varieties, and the breeding of new heat-resistant cultivars are crucial for preventing losses in rice production. Understanding the physiological and genetic mechanisms of heat tolerance is essential for developing new rice varieties that are resilient and productive under diverse environmental conditions. Additionally, adopting resistant rice genotypes can help reduce yield losses even further. While increasing plant tolerance to heat is crucial, ensuring that this does not compromise yield or quality is vital.

High temperatures negatively affect all rice growth stages, with the flowering stage (anthesis) being the most vulnerable. Combining plant hormones and exogenous osmoprotectants can trigger a temporary acclimation response in plants, helping them withstand high-temperature stress. This "priming" approach, especially when applied at critical growth stages, has shown promise in reducing the detrimental reactions to heat shock. Although some research has been conducted on the use of plant hormones, additional studies are required to fully understand their effectiveness in reducing heat stress. This information will help develop optimal strategies for protecting crops under rising temperatures. Adapting and implementing various modern techniques, including climate modelling tools and GIS, requires a combination of methodologies. These methods include molecular approaches such as genomics, proteomics, and transcriptomics. Considering trait dependence and variation is vital, as specific genotypes show tolerance only in certain environmental conditions. Further investigations are needed to comprehensively examine the genetic diversity of rice by discerning the physiological processes that support rice production amidst the changing environment and assessing the efficacy of heat stress management strategies. By implementing these strategies, farmers can effectively manage heat shock and mitigate its impact on rice production while working toward achieving sustainable and resilient agricultural systems. Continued research, collaboration, and investments are crucial in developing and implementing these strategies to ensure a secure and reliable rice supply in the face of climate change-induced heat shock in rice.

## CRediT authorship contribution statement

**Mohammad Mobarak Hossain:** Writing – review & editing, Writing – original draft, Software, Formal analysis, Data curation, Conceptualization. **Sharif Ahmed:** Writing – review & editing, Writing – original draft, Data curation, Conceptualization. **Mohammad Saiful Alam:** Writing – review & editing, Writing – original draft, Conceptualization. **Akbar Hossain:** Writing – review & editing, Writing – original draft, Supervision, Software, Formal analysis, Data curation, Conceptualization.

## Data availability statement

While not accessible to the general public, the corresponding author may provide access to the data upon request.

## Declaration of competing interest

The authors declare that they have no known competing financial interests or personal relationships that could have appeared to influence the work reported in this paper.
